# How COVID-19 Impacts Chinese Travelers’ Mobility Decision-Making Processes: A Bayesian Network Model

**DOI:** 10.1007/978-3-030-65785-7_53

**Published:** 2020-11-28

**Authors:** Junyi Wang, Xueting Zhai, Qiuju Luo

**Affiliations:** 1 Department for Informatics, Technical University of Munich, Garching bei München, Bayern Germany; 2 Smart Tourism Education Platform (STEP) College of Hotel and Tourism Management, Kyung Hee University, Seoul, Korea (Republic of); 3 Department of Tourism and Service Management, MODUL University Vienna, Vienna, Wien Austria; Sun Yat-Sen University, Zhuhai, 519082 People’s Republic of China

**Keywords:** COVID-19, Traveler mobility, Decision-Making process, Bayesian network model

## Abstract

The outbreak of the COVID-19 pandemic has a multi-faceted impact on the mobility of travelers. Current research has not yet explained the internal mechanisms of travelers’ mobility changes during the pandemic. The Bayesian network is considered to be an effective method to describe the causality between the factors and output of a system. Thus, this paper established a Bayesian network model to analyze the impact of COVID-19 on Chinese travelers’ mobility decision–making processes. The model for the traveler mobility decision-making process is built on both a qualitative and quantitative analysis of travelers’ self-narration articles. Results show that official information, traffic information, family structure, and social interaction networks are the key factors affecting Chinese travelers’ mobility.

## Introduction

The large scale of travelers’ flow has been a major risk during the COVID-19 pandemic. Travelers’ emotions and mobile behaviors have become more changeable and anomalous, even resulting in disorder flow or chaos due to fear and uncertainty. Some factors that impact travelers’ mobility decision making during the COVID-19 pandemic include government policies (e.g. border closures), accessibility (e.g. flight cancellations), and social media information. In addition to such general factors, *family and culture* are influencers that are particularly found in Chinese travelers’ mobility patterns. Since the COVID-19 outbreak in China happened during the Chinese Spring Festival—the most important traditional festival in China, when people are expected to return to their hometowns—Chinese travelers’ mobility decisions are not only affected by their own needs but also their families, customs, and morals. Therefore, it is interesting and significant to explore the decision-making process concerning Chinese travelers’ mobility during COVID-19.

Travelers’ mobility and decision making have always been hot topics in tourism research [1, 2]. Current research has demonstrated that the traveler mobility decision-making process is complex, determined by a wide range of factors stemming from the economy, sociology, geography, and psychology [3]. Thus, it is still a challenge to predict how travelers’ mobility behaviors and their underlying mechanisms will change. Undoubtfully, COVID-19 will impact travelers’ mobility decisions, but how?

Certain studies have constructed spatiotemporal diffusion models to reveal the relationship between public flow and the spread of disease [4]. Some have discussed the impact of the epidemic on traveler mobility behavior, thus revealing particular abnormal mobility behaviors [5, 6]. However, most of the existing research has been based on epidemiological statistics, which may visualize the general flow while failing to explain the underlying mechanisms. Moreover, current research has not explained the behavior changes of heterogeneous travelers during the pandemic.

To fill the gaps, this study used the Bayesian network method to establish a causal model of Chinese travelers’ mobility decision-making processes during the COVID-19 pandemic and identify key influential factors.

## Method

### Data Collection

We collected 60 non-fictional self-narration articles released on WeChat—one of the most popular social media platforms in China—from Jan. 28, 2020, to Jun. 4, 2020. In these sampled articles, the authors recalled and stated their mobility routines from Jan. 18, 2020, to Mar. 31, 2020. This exact time period covers the outbreak and spread of COVID-19 in China [7], including two important Chinese traditional festivals: the Spring Festival on Jan. 25, 2020 and the Lantern Festival on Feb. 8, 2020. This period is typically a peak of mobility for Chinese travelers because it is a traditional custom for Chinese people to return to their hometowns during the Spring Festival until the Lantern Festival. Thus, it is interesting to reveal how Chinese travelers negotiated information about the pandemic, travel risks, and cultural customs in their traveling decisions. These articles involved 63 individuals’ mobile behaviors in total, including 24 men and 39 women. In accordance with their publication time, the self-narration articles were divided into two sample sets, with 30 articles each.

### Bayesian Network Model

The Bayesian network is a directed acyclic graphical model that represents the probability dependence between variables [8]. Bayesian network modeling consists of the following steps: (1) Structure determination. We used NVivo 12 software to code the self-narration articles in Sample 1 to identify the elements and topics that affect travelers’ mobile decisions. Then, cross-comparison was conducted by the three authors to discuss and recode the inconsistent codes. Finally, we identified the specific relationship between the elements and themes and constructed a directed acyclic network structure. (2) Parameter determination. We used GeNIe software to draw a Bayesian network model. Then, we employed experts to assign the initial conditional probability. (3) Parameter learning. We coded the elements in Sample 2 in accordance with the coding rules as Sample 1 to establish a quantitative data set. Then, based on the Bayesian network built in Step 2 and the quantitative data set, we used the EM algorithm method to adjust the model. (4) Sensitivity analysis. We used sensitivity analysis to evaluate the Bayesian network model and identify key influential factors.

## Bayesian Network Modeling

### Structure Determination

Six main factors and 16 specific nodes were identified to construct a Bayesian network model of travelers’ mobility changes (Table 1).

**Table 1.** Variables of traveler mobility changes

Factors	Variables	Variables States
Information Sources	Official Information	Departure place, Destination, Channel, Other places
	Word of Mouth	Cyberspace, Physical space
	Traffic Information	Yes, No
	Epidemic Information	Yes, No
Social Environment	Destination Environment	Relatives and Friends, Traveler, Residents
	Departure Place Environment	Relatives and Friends, Traveler, Residents
Personal Factors	Family Structure	Core, Stem, Joint
	Habits	Personal, Family, Social
	Prior Knowledge	Experience, Knowledge
	Social Interaction Network	Close, Distant
Emotional Experience	Emotion State	Positive, Negative
	Emotional Arousal	High, Medium, Low
Decision Process	Decision Orientation	For Self, Others, Group
	Risk Perception	Severity, Susceptibility
Behavior Outcome	Mobility Behavior	Increase, No Change, Decrease
	Self-Protection Behavior	Increase, No Change, Decrease

**Information Sources.** After the outbreak of COVID-19, complex and uncertain information directly and immediately affected travelers’ mobile decisions, resulting in sudden route changes. For example, *“…a friend sent me a road closure notice in Tianmen, Hubei. Out of intuition, I decided to pack my things immediately, set off after New Year’s Eve dinner, and returned early.”*

**Social Environment.** Social environment is a relatively stable external influence in mobile decision making [9]. However, during the COVID-19 pandemic, the social environment deviated from the normal state, making it difficult to predict. The mobile behavior of travelers is influenced by—and sometimes contended with—the social environment and Chinese culture. For example, “*According to the custom of my hometown, we must visit our relatives and friends on the first day of the first lunar month. My uncle did not think it necessary to be so careful (regarding COVID*-*19), while my mother stood firm on her principles and refused to let anyone visit her.”*

**Personal Factors.** Previous experience, family structure, social interaction networks and customs are the main personal factors that affect traveler mobility. Travelers’ previous experiences not only affect their risk perception but also arouse their previous emotional experience, consequently strengthening self-protection and impacting mobility decisions. For example, *“…those experiences (during SARS) are vivid. So, I…cancelled my wife’s and children’s train tickets and returned to Wuhan alone.”* Travelers also consider their family structure, customs, and relationships when making decisions. For example, *“When the elderly pass away, it is an especially important and serious custom for relatives to worship on the first day of the new year. A sudden cancellation is too abrupt. I dare not make that decision.”*

**Emotional Experience.** Most sampled travelers experienced negative emotions during the COVID-19 pandemic with a high or medium level of emotional arousal. Panic and worry are the driving forces leading to sudden changes of travelers’ mobility behavior. For example, *“There are two old people and one young child in my hometown. What are their conditions? These worries have made me restless for several days. On January 22, I returned home from Shenzhen alone.”*

**Decision Process.** The decision-making process includes two factors that are key in ultimately determining if and how the mobility decision changes: decision orientation and risk perception. Travelers show different attitudes and preferences when they make decisions taking various aspects into consideration [10]. For example, *“COVID*-*19 became serious at the time, and I felt that traveling to the UK might cause discomfort to the locals to some extent, so I asked for a refund for my ticket.”*

### Parameter Learning

**Fig. 1.**
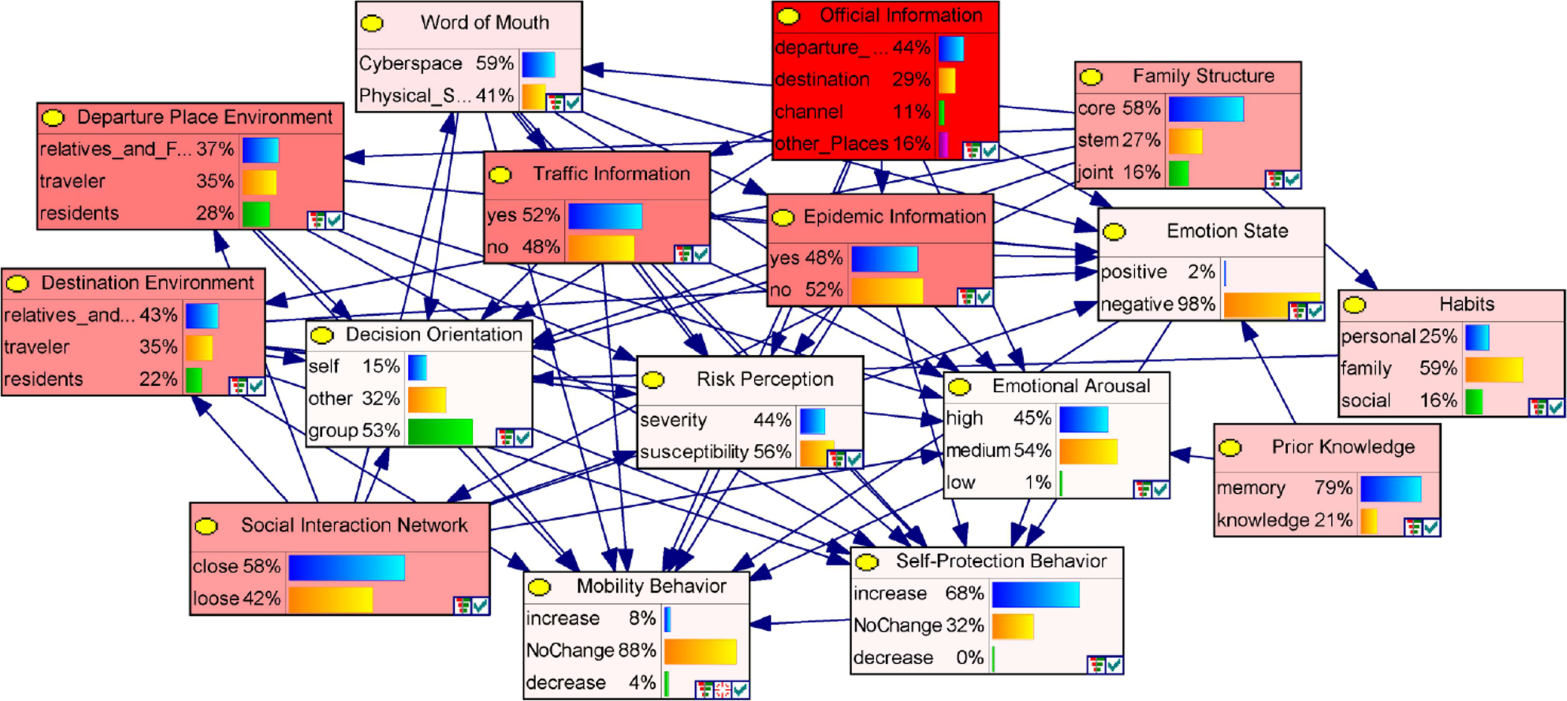
The Bayesian network model and sensitive analysis of travelers’ decision making

Combining both expert assignments and quantitative data, we assigned the confidence of the original parameters to 30, equal to the sample size. Then we used the EM algorithm method to conduct parameter learning to accommodate the missing data. Genie2.0 was used for Bayesian network parameter learning. Finally, a Bayesian model was built to simulate travelers’ mobility decision-making processes (Fig. 1).

### Sensitivity Analysis

Since the travelers’ decision-making behaviors are affected by multiple factors, sensitivity analysis was used to explore the influence of each factor (set) and then figure out the most important ones. Figure 1 showed that information sources are the most important factor impacting changes in travelers’ mobility behavior. Among them, official information has the highest sensitivity value, which means that travelers’ mobility behavior is highly sensitive to the official information released. Social environment and personal factors also greatly impacted traveler mobility.

**Fig. 2.**
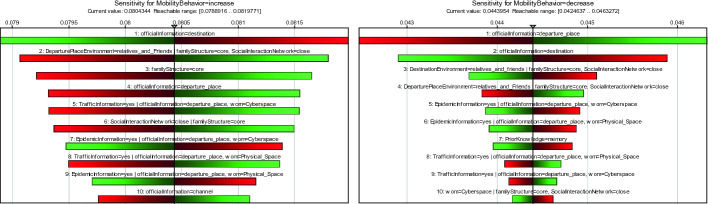
Sensitivity tornado of mobility behavior

Further, we drew a sensitivity tornado of the increased and decreased states of mobility behavior and compared the sensitivity of each factor (Fig. 2). The top five factors (set) that may enhance the possibility of travelers to change their mobility include official information, departure place environment, family structure, traffic information, and social interaction network. While the official information from the destination may decrease the possibility of travelers increasing their mobility, official information from the departure place might enhance the possibility of travelers reducing their mobility.

## Conclusions

This research constructed a Bayesian network model to simulate Chinese travelers’ mobility decision-making processes during the COVID-19 pandemic. The Bayesian network is widely applied to explain highly complex phenomena and decision-making processes. Results show that official information, traffic information, family structure and social interaction networks are the key factors (set) affecting the mobility of travelers. In addition, information sources and social environment not only directly affect the changes of travelers’ mobility, but they also have indirect effects through travelers’ decision processes and emotions. As a result, Chinese travelers’ mobility decision-making processes are not only influenced by information about the pandemic but also culture, customs, and morals.
